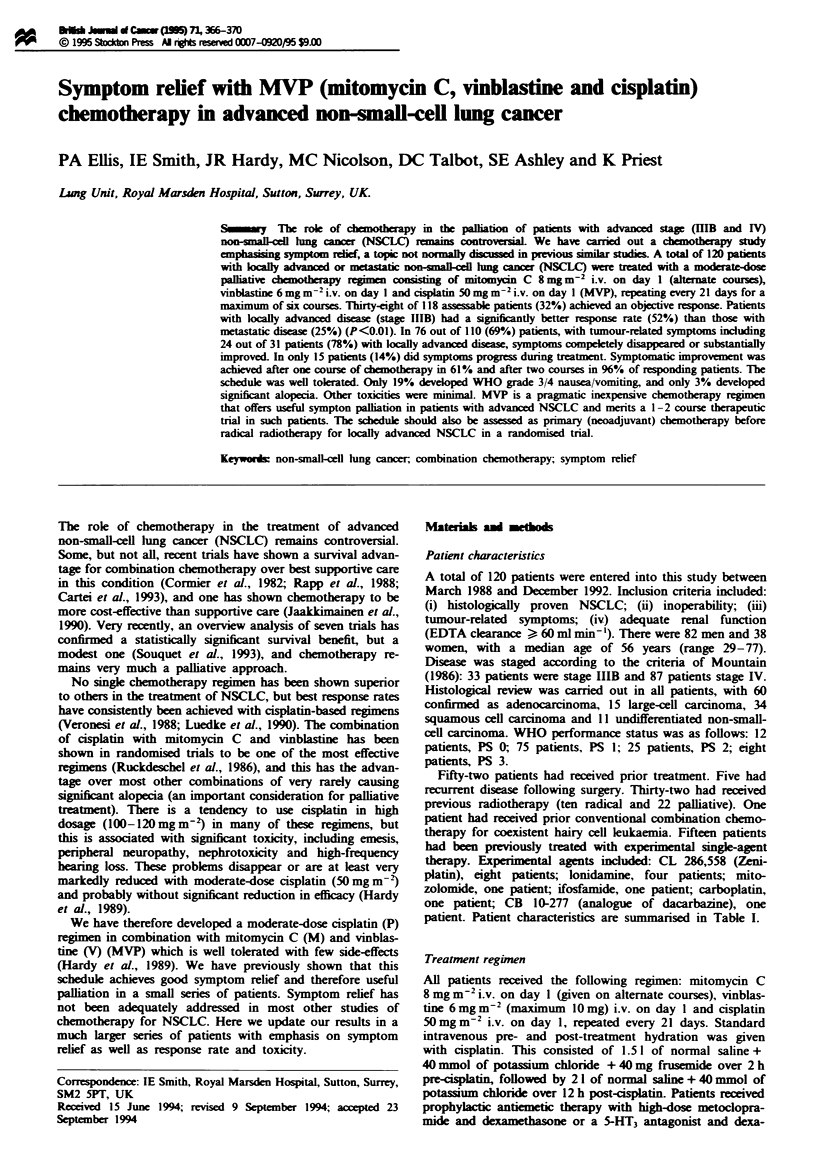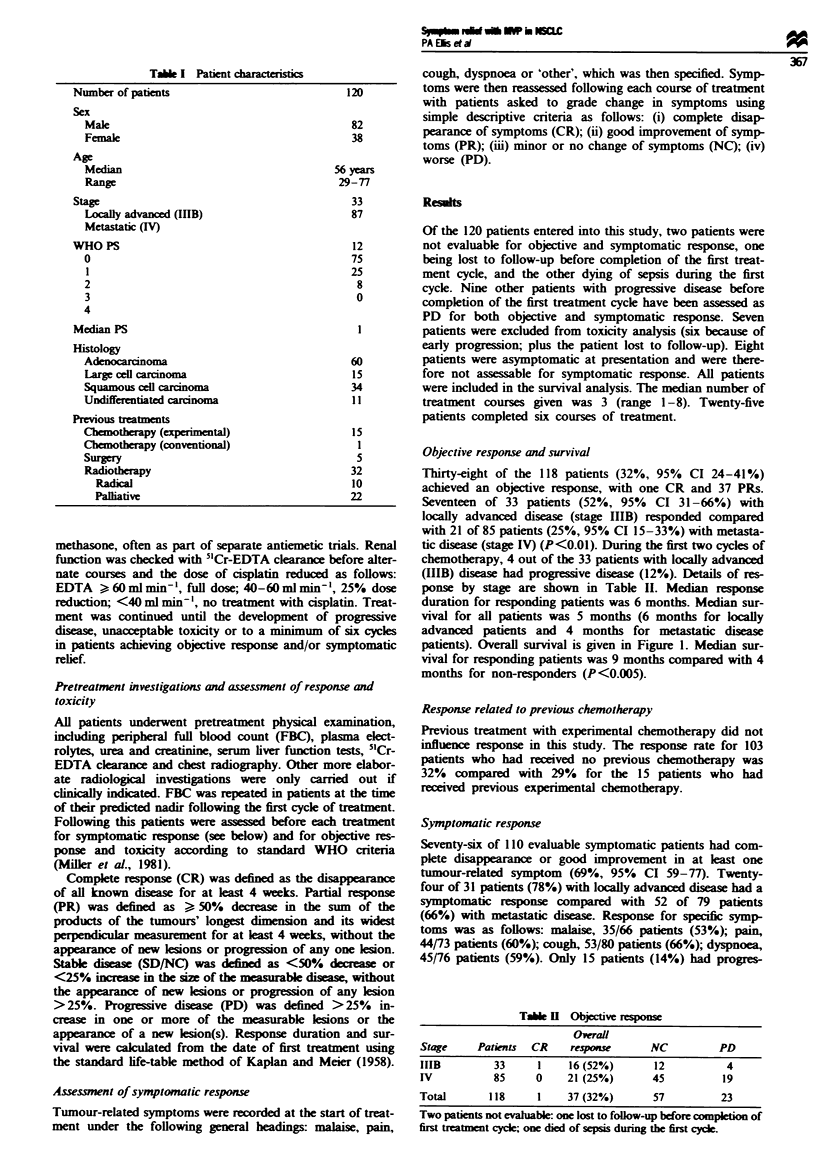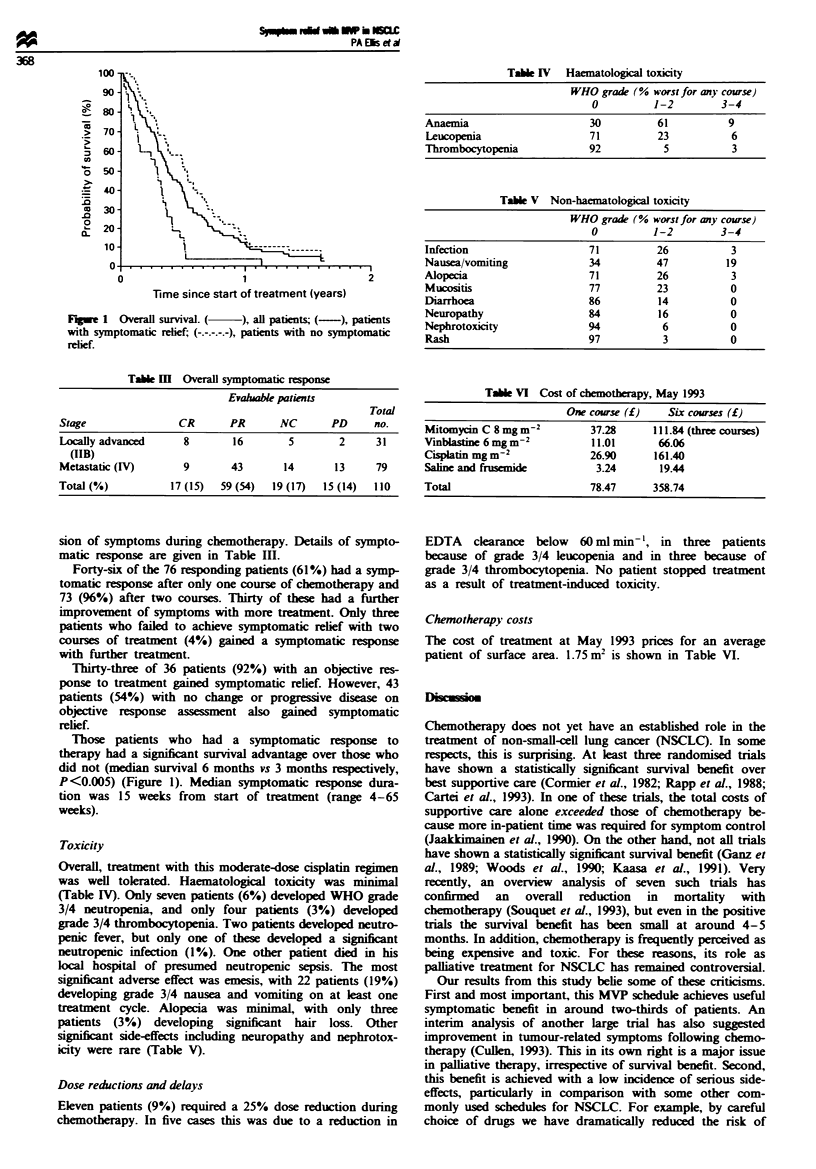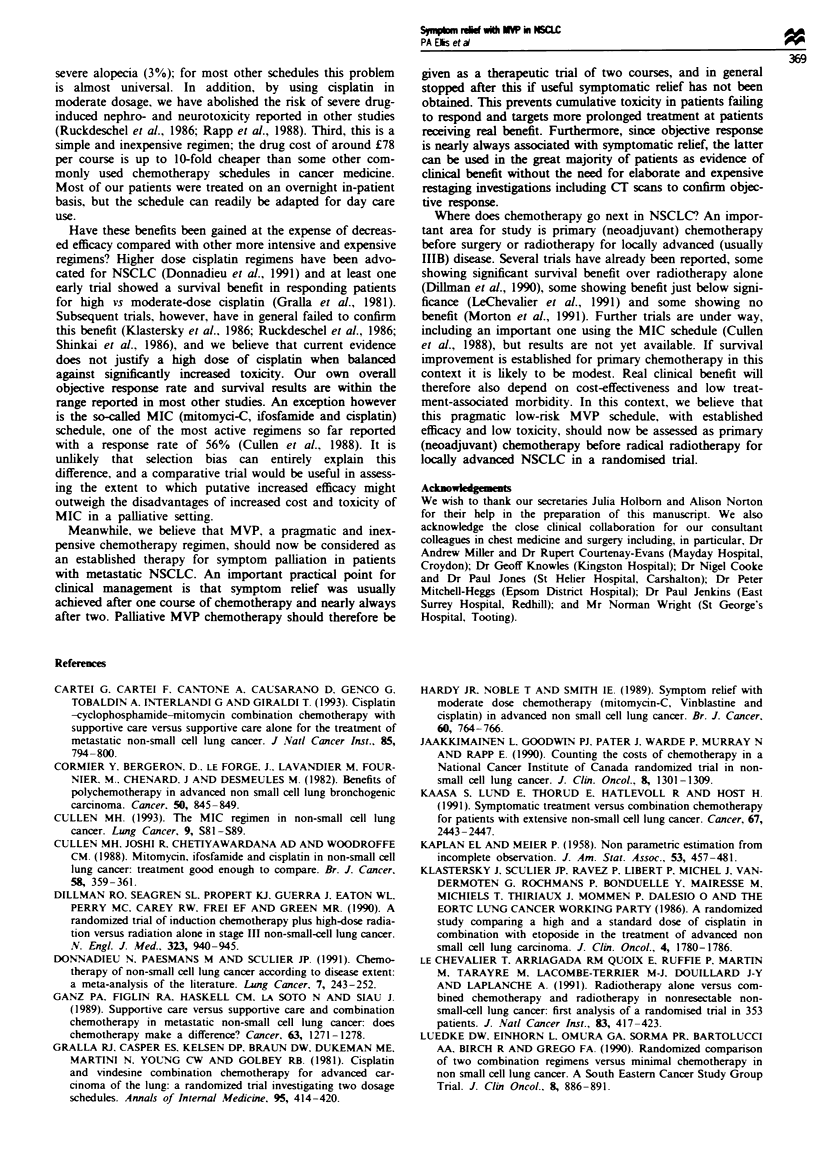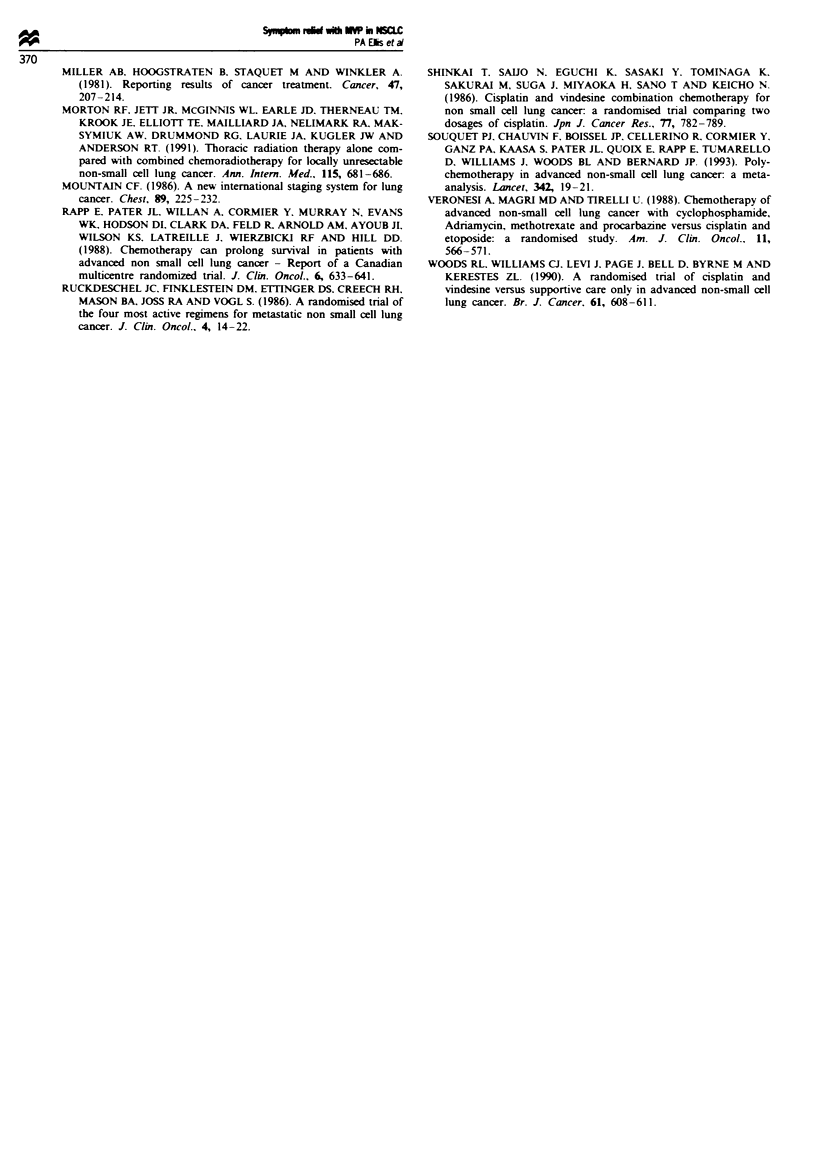# Symptom relief with MVP (mitomycin C, vinblastine and cisplatin) chemotherapy in advanced non-small-cell lung cancer.

**DOI:** 10.1038/bjc.1995.74

**Published:** 1995-02

**Authors:** P. A. Ellis, I. E. Smith, J. R. Hardy, M. C. Nicolson, D. C. Talbot, S. E. Ashley, K. Priest

**Affiliations:** Lung Unit, Royal Marsden Hospital, Sutton, Surrey, UK.

## Abstract

The role of chemotherapy in the palliation of patients with advanced stage (IIIB and IV non-small-cell lung cancer (NSCLC) remains controversial. We have carried out a chemotherapy study emphasising symptom relief, a topic not normally discussed in previous similar studies. A total of 120 patients with locally advanced or metastatic non-small-cell lung cancer (NSCLC) were treated with a moderate-dose palliative chemotherapy regimen consisting of mitomycin C 8 mg m-2 i.v. on day 1 (alternate courses), vinblastine 6 mg m-2 i.v. on day 1 and cisplatin 50 mg m-2 i.v. on day 1 (MVP), repeating every 21 days for a maximum of six courses. Thirty-eight of 118 assessable patients (32%) achieved an objective response. Patients with locally advanced disease (stage IIIB) had a significantly better response rate (52%) than those with metastatic disease (25%) (P < 0.01). In 76 out of 110 (69%) patients, with tumour-related symptoms including 24 out of 31 patients (78%) with locally advanced disease, symptoms completely disappeared or substantially improved. In only 15 patients (14%) did symptoms progress during treatment. Symptomatic improvement was achieved after one course of chemotherapy in 61% and after two courses in 96% of responding patients. The schedule was well tolerated. Only 19% developed WHO grade 3/4 nausea/vomiting, and only 3% developed significant alopecia. Other toxicities were minimal. MVP is a pragmatic inexpensive chemotherapy regimen that offers useful symptom palliation in patients with advanced NSCLC and merits a 1-2 course therapeutic trial in such patients. The schedule should also be assessed as primary (neoadjuvant) chemotherapy before radical radiotherapy for locally advanced NSCLC in a randomised trial.


### Symptom relief with MVP (mitomycin C, vinblastine and cisplatin) chemotherapy in advanced non-small-cell lung cancer

PA Ellis, IE Smith, JR Hardy, MC Nicolson, DC Talbot, SE Ashley and K Priest

*Lung Unit, Royal Marsden Hospital, Sutton, Surrey, UK.*

**Summary** The role of chemotherapy in the palliation of patients with advanced stage (IIIB and IV) non-small-cell lung cancer (NSCLC) remains controversial. We have carried out a chemotherapy study emphasising symptom relief, a topic not normally discussed in previous similar studies. A total of 120 patients with locally advanced or metastatic non-small-cell lung cancer (NSCLC) were treated with a moderate-dose palliative chemotherapy regimen consisting of mitomycin C 8 mg m$^{-2}$ i.v. on day 1 (alternate courses), vinblastine 6 mg m$^{-2}$ i.v. on day 1 and cisplatin 50 mg m$^{-2}$ i.v. on day 1 (MVP), repeating every 21 days for a maximum of six courses. Thirty-eight of 118 assessable patients (32%) achieved an objective response. Patients with locally advanced disease (stage IIIB) had a significantly better response rate (52%) than those with metastatic disease (25%) ($P<0.01$). In 76 out of 110 (69%) patients, with tumour-related symptoms including 24 out of 31 patients (78%) with locally advanced disease, symptoms compeletely disappeared or substantially improved. In only 15 patients (14%) did symptoms progress during treatment. Symptomatic improvement was achieved after one course of chemotherapy in 61% and after two courses in 96% of responding patients. The schedule was well tolerated. Only 19% developed WHO grade 3/4 nausea/vomiting, and only 3% developed significant alopecia. Other toxicities were minimal. MVP is a pragmatic inexpensive chemotherapy regimen that offers useful sympton palliation in patients with advanced NSCLC and merits a 1–2 course therapeutic trial in such patients. The schedule should also be assessed as primary (neoadjuvant) chemotherapy before radical radiotherapy for locally advanced NSCLC in a randomised trial.

**Keywords:** non-small-cell lung cancer; combination chemotherapy; symptom relief

The role of chemotherapy in the treatment of advanced non-small-cell lung cancer (NSCLC) remains controversial. Some, but not all, recent trials have shown a survival advantage for combination chemotherapy over best supportive care in this condition (Cormier *et al.*, 1982; Rapp *et al.*, 1988; Cartei *et al.*, 1993), and one has shown chemotherapy to be more cost-effective than supportive care (Jaakkimainen *et al.*, 1990). Very recently, an overview analysis of seven trials has confirmed a statistically significant survival benefit, but a modest one (Souquet *et al.*, 1993), and chemotherapy remains very much a palliative approach.

No single chemotherapy regimen has been shown superior to others in the treatment of NSCLC, but best response rates have consistently been achieved with cisplatin-based regimens (Veronesi *et al.*, 1988; Luedke *et al.*, 1990). The combination of cisplatin with mitomycin C and vinblastine has been shown in randomised trials to be one of the most effective regimens (Ruckdeschel *et al.*, 1986), and this has the advantage over most other combinations of very rarely causing significant alopecia (an important consideration for palliative treatment). There is a tendency to use cisplatin in high dosage (100–120 mg m$^{-2}$) in many of these regimens, but this is associated with significant toxicity, including emesis, peripheral neuropathy, nephrotoxicity and high-frequency hearing loss. These problems disappear or are at least very markedly reduced with moderate-dose cisplatin (50 mg m$^{-2}$) and probably without significant reduction in efficacy (Hardy *et al.*, 1989).

We have therefore developed a moderate-dose cisplatin (P) regimen in combination with mitomycin C (M) and vinblastine (V) (MVP) which is well tolerated with few side-effects (Hardy *et al.*, 1989). We have previously shown that this schedule achieves good symptom relief and therefore useful palliation in a small series of patients. Symptom relief has not been adequately addressed in most other studies of chemotherapy for NSCLC. Here we update our results in a much larger series of patients with emphasis on symptom relief as well as response rate and toxicity.

Correspondence: IE Smith, Royal Marsden Hospital, Sutton, Surrey, SM2 5PT, UK
Received 15 June 1994; revised 9 September 1994; accepted 23 September 1994

### Materials and methods

### *Patient characteristics*

A total of 120 patients were entered into this study between March 1988 and December 1992. Inclusion criteria included: (i) histologically proven NSCLC; (ii) inoperability; (iii) tumour-related symptoms; (iv) adequate renal function (EDTA clearance $\geqslant 60$ ml min$^{-1}$). There were 82 men and 38 women, with a median age of 56 years (range 29–77). Disease was staged according to the criteria of Mountain (1986): 33 patients were stage IIIB and 87 patients stage IV. Histological review was carried out in all patients, with 60 confirmed as adenocarcinoma, 15 large-cell carcinoma, 34 squamous cell carcinoma and 11 undifferentiated non-small-cell carcinoma. WHO performance status was as follows: 12 patients, PS 0; 75 patients, PS 1; 25 patients, PS 2; eight patients, PS 3.

Fifty-two patients had received prior treatment. Five had recurrent disease following surgery. Thirty-two had received previous radiotherapy (ten radical and 22 palliative). One patient had received prior conventional combination chemotherapy for coexistent hairy cell leukaemia. Fifteen patients had been previously treated with experimental single-agent therapy. Experimental agents included: CL 286,558 (Zeniplatin), eight patients; lonidamine, four patients; mitozolomide, one patient; ifosfamide, one patient; carboplatin, one patient; CB 10-277 (analogue of dacarbazine), one patient. Patient characteristics are summarised in Table I.

### *Treatment regimen*

All patients received the following regimen: mitomycin C 8 mg m$^{-2}$ i.v. on day 1 (given on alternate courses), vinblastine 6 mg m$^{-2}$ (maximum 10 mg) i.v. on day 1 and cisplatin 50 mg m$^{-2}$ i.v. on day 1, repeated every 21 days. Standard intravenous pre- and post-treatment hydration was given with cisplatin. This consisted of 1.5 l of normal saline + 40 mmol of potassium chloride + 40 mg frusemide over 2 h pre-cisplatin, followed by 2 l of normal saline + 40 mmol of potassium chloride over 12 h post-cisplatin. Patients received prophylactic antiemetic therapy with high-dose metoclopramide and dexamethasone or a 5-$HT_3$ antagonist and dexa-

**Table I** Patient characteristics

| | |
|---|---|
| Number of patients | 120 |
| Sex | |
| Male | 82 |
| Female | 38 |
| Age | |
| Median | 56 years |
| Range | 29–77 |
| Stage | 33 |
| Locally advanced (IIIB) | 87 |
| Metastatic (IV) | |
| WHO PS | 12 |
| 0 | 75 |
| 1 | 25 |
| 2 | 8 |
| 3 | 0 |
| 4 | |
| Median PS | 1 |
| Histology | |
| Adenocarcinoma | 60 |
| Large cell carcinoma | 15 |
| Squamous cell carcinoma | 34 |
| Undifferentiated carcinoma | 11 |
| Previous treatments | |
| Chemotherapy (experimental) | 15 |
| Chemotherapy (conventional) | 1 |
| Surgery | 5 |
| Radiotherapy | 32 |
| Radical | 10 |
| Palliative | 22 |

methasone, often as part of separate antiemetic trials. Renal function was checked with $^{51}$Cr-EDTA clearance before alternate courses and the dose of cisplatin reduced as follows: EDTA $\geqslant 60$ ml min$^{-1}$, full dose; 40–60 ml min$^{-1}$, 25% dose reduction; $<40$ ml min$^{-1}$, no treatment with cisplatin. Treatment was continued until the development of progressive disease, unacceptable toxicity or to a minimum of six cycles in patients achieving objective response and/or symptomatic relief.

*Pretreatment investigations and assessment of response and toxicity*

All patients underwent pretreatment physical examination, including peripheral full blood count (FBC), plasma electrolytes, urea and creatinine, serum liver function tests, $^{51}$Cr-EDTA clearance and chest radiography. Other more elaborate radiological investigations were only carried out if clinically indicated. FBC was repeated in patients at the time of their predicted nadir following the first cycle of treatment. Following this patients were assessed before each treatment for symptomatic response (see below) and for objective response and toxicity according to standard WHO criteria (Miller *et al.*, 1981).

Complete response (CR) was defined as the disappearance of all known disease for at least 4 weeks. Partial response (PR) was defined as $\geqslant 50\%$ decrease in the sum of the products of the tumours' longest dimension and its widest perpendicular measurement for at least 4 weeks, without the appearance of new lesions or progression of any one lesion. Stable disease (SD/NC) was defined as $<50\%$ decrease or $<25\%$ increase in the size of the measurable disease, without the appearance of new lesions or progression of any lesion $>25\%$. Progressive disease (PD) was defined $>25\%$ increase in one or more of the measurable lesions or the appearance of a new lesion(s). Response duration and survival were calculated from the date of first treatment using the standard life-table method of Kaplan and Meier (1958).

*Assessment of symptomatic response*

Tumour-related symptoms were recorded at the start of treatment under the following general headings: malaise, pain, cough, dyspnoea or 'other', which was then specified. Symptoms were then reassessed following each course of treatment with patients asked to grade change in symptoms using simple descriptive criteria as follows: (i) complete disappearance of symptoms (CR); (ii) good improvement of symptoms (PR); (iii) minor or no change of symptoms (NC); (iv) worse (PD).

### Results

Of the 120 patients entered into this study, two patients were not evaluable for objective and symptomatic response, one being lost to follow-up before completion of the first treatment cycle, and the other dying of sepsis during the first cycle. Nine other patients with progressive disease before completion of the first treatment cycle have been assessed as PD for both objective and symptomatic response. Seven patients were excluded from toxicity analysis (six because of early progression; plus the patient lost to follow-up). Eight patients were asymptomatic at presentation and were therefore not assessable for symptomatic response. All patients were included in the survival analysis. The median number of treatment courses given was 3 (range 1–8). Twenty-five patients completed six courses of treatment.

*Objective response and survival*

Thirty-eight of the 118 patients (32%, 95% CI 24–41%) achieved an objective response, with one CR and 37 PRs. Seventeen of 33 patients (52%, 95% CI 31–66%) with locally advanced disease (stage IIIB) responded compared with 21 of 85 patients (25%, 95% CI 15–33%) with metastatic disease (stage IV) ($P<0.01$). During the first two cycles of chemotherapy, 4 out of the 33 patients with locally advanced (IIIB) disease had progressive disease (12%). Details of response by stage are shown in Table II. Median response duration for responding patients was 6 months. Median survival for all patients was 5 months (6 months for locally advanced patients and 4 months for metastatic disease patients). Overall survival is given in Figure 1. Median survival for responding patients was 9 months compared with 4 months for non-responders ($P<0.005$).

*Response related to previous chemotherapy*

Previous treatment with experimental chemotherapy did not influence response in this study. The response rate for 103 patients who had received no previous chemotherapy was 32% compared with 29% for the 15 patients who had received previous experimental chemotherapy.

*Symptomatic response*

Seventy-six of 110 evaluable symptomatic patients had complete disappearance or good improvement in at least one tumour-related symptom (69%, 95% CI 59–77). Twenty-four of 31 patients (78%) with locally advanced disease had a symptomatic response compared with 52 of 79 patients (66%) with metastatic disease. Response for specific symptoms was as follows: malaise, 35/66 patients (53%); pain, 44/73 patients (60%); cough, 53/80 patients (66%); dyspnoea, 45/76 patients (59%). Only 15 patients (14%) had progres-

**Table II** Objective response

| *Stage* | *Patients* | *CR* | *Overall response* | *NC* | *PD* |
|---|---|---|---|---|---|
| IIIB | 33 | 1 | 16 (52%) | 12 | 4 |
| IV | 85 | 0 | 21 (25%) | 45 | 19 |
| Total | 118 | 1 | 37 (32%) | 57 | 23 |

Two patients not evaluable: one lost to follow-up before completion of first treatment cycle; one died of sepsis during the first cycle.

**Figure 1** Overall survival. (———), all patients; (——), patients with symptomatic relief; (-.-.-.-), patients with no symptomatic relief.

**Table III** Overall symptomatic response

| Stage | *Evaluable patients* CR | PR | NC | PD | *Total no.* |
|---|---|---|---|---|---|
| Locally advanced (IIB) | 8 | 16 | 5 | 2 | 31 |
| Metastatic (IV) | 9 | 43 | 14 | 13 | 79 |
| Total (%) | 17 (15) | 59 (54) | 19 (17) | 15 (14) | 110 |

**Table IV** Haematological toxicity

| | *WHO grade (% worst for any course)* 0 | 1–2 | 3–4 |
|---|---|---|---|
| Anaemia | 30 | 61 | 9 |
| Leucopenia | 71 | 23 | 6 |
| Thrombocytopenia | 92 | 5 | 3 |

**Table V** Non-haematological toxicity

| | *WHO grade (% worst for any course)* 0 | 1–2 | 3–4 |
|---|---|---|---|
| Infection | 71 | 26 | 3 |
| Nausea/vomiting | 34 | 47 | 19 |
| Alopecia | 71 | 26 | 3 |
| Mucositis | 77 | 23 | 0 |
| Diarrhoea | 86 | 14 | 0 |
| Neuropathy | 84 | 16 | 0 |
| Nephrotoxicity | 94 | 6 | 0 |
| Rash | 97 | 3 | 0 |

**Table VI** Cost of chemotherapy, May 1993

| | *One course (£)* | *Six courses (£)* |
|---|---|---|
| Mitomycin C 8 mg $m^{-2}$ | 37.28 | 111.84 (three courses) |
| Vinblastine 6 mg $m^{-2}$ | 11.01 | 66.06 |
| Cisplatin mg $m^{-2}$ | 26.90 | 161.40 |
| Saline and frusemide | 3.24 | 19.44 |
| Total | 78.47 | 358.74 |

sion of symptoms during chemotherapy. Details of symptomatic response are given in Table III.

Forty-six of the 76 responding patients (61%) had a symptomatic response after only one course of chemotherapy and 73 (96%) after two courses. Thirty of these had a further improvement of symptoms with more treatment. Only three patients who failed to achieve symptomatic relief with two courses of treatment (4%) gained a symptomatic response with further treatment.

Thirty-three of 36 patients (92%) with an objective response to treatment gained symptomatic relief. However, 43 patients (54%) with no change or progressive disease on objective response assessment also gained symptomatic relief.

Those patients who had a symptomatic response to therapy had a significant survival advantage over those who did not (median survival 6 months *vs* 3 months respectively, $P<0.005$) (Figure 1). Median symptomatic response duration was 15 weeks from start of treatment (range 4–65 weeks).

### *Toxicity*

Overall, treatment with this moderate-dose cisplatin regimen was well tolerated. Haematological toxicity was minimal (Table IV). Only seven patients (6%) developed WHO grade 3/4 neutropenia, and only four patients (3%) developed grade 3/4 thrombocytopenia. Two patients developed neutropenic fever, but only one of these developed a significant neutropenic infection (1%). One other patient died in his local hospital of presumed neutropenic sepsis. The most significant adverse effect was emesis, with 22 patients (19%) developing grade 3/4 nausea and vomiting on at least one treatment cycle. Alopecia was minimal, with only three patients (3%) developing significant hair loss. Other significant side-effects including neuropathy and nephrotoxicity were rare (Table V).

### *Dose reductions and delays*

Eleven patients (9%) required a 25% dose reduction during chemotherapy. In five cases this was due to a reduction in EDTA clearance below 60 ml $min^{-1}$, in three patients because of grade 3/4 leucopenia and in three because of grade 3/4 thrombocytopenia. No patient stopped treatment as a result of treatment-induced toxicity.

### *Chemotherapy costs*

The cost of treatment at May 1993 prices for an average patient of surface area. 1.75 $m^2$ is shown in Table VI.

### Discussion

Chemotherapy does not yet have an established role in the treatment of non-small-cell lung cancer (NSCLC). In some respects, this is surprising. At least three randomised trials have shown a statistically significant survival benefit over best supportive care (Cormier *et al.*, 1982; Rapp *et al.*, 1988; Cartei *et al.*, 1993). In one of these trials, the total costs of supportive care alone *exceeded* those of chemotherapy because more in-patient time was required for symptom control (Jaakkimainen *et al.*, 1990). On the other hand, not all trials have shown a statistically significant survival benefit (Ganz *et al.*, 1989; Woods *et al.*, 1990; Kaasa *et al.*, 1991). Very recently, an overview analysis of seven such trials has confirmed an overall reduction in mortality with chemotherapy (Souquet *et al.*, 1993), but even in the positive trials the survival benefit has been small at around 4–5 months. In addition, chemotherapy is frequently perceived as being expensive and toxic. For these reasons, its role as palliative treatment for NSCLC has remained controversial.

Our results from this study belie some of these criticisms. First and most important, this MVP schedule achieves useful symptomatic benefit in around two-thirds of patients. An interim analysis of another large trial has also suggested improvement in tumour-related symptoms following chemotherapy (Cullen, 1993). This in its own right is a major issue in palliative therapy, irrespective of survival benefit. Second, this benefit is achieved with a low incidence of serious side-effects, particularly in comparison with some other commonly used schedules for NSCLC. For example, by careful choice of drugs we have dramatically reduced the risk of severe alopecia (3%); for most other schedules this problem is almost universal. In addition, by using cisplatin in moderate dosage, we have abolished the risk of severe drug-induced nephro- and neurotoxicity reported in other studies (Ruckdeschel *et al.*, 1986; Rapp *et al.*, 1988). Third, this is a simple and inexpensive regimen; the drug cost of around £78 per course is up to 10-fold cheaper than some other commonly used chemotherapy schedules in cancer medicine. Most of our patients were treated on an overnight in-patient basis, but the schedule can readily be adapted for day care use.

Have these benefits been gained at the expense of decreased efficacy compared with other more intensive and expensive regimens? Higher dose cisplatin regimens have been advocated for NSCLC (Donnadieu *et al.*, 1991) and at least one early trial showed a survival benefit in responding patients for high *vs* moderate-dose cisplatin (Gralla *et al.*, 1981). Subsequent trials, however, have in general failed to confirm this benefit (Klastersky *et al.*, 1986; Ruckdeschel *et al.*, 1986; Shinkai *et al.*, 1986), and we believe that current evidence does not justify a high dose of cisplatin when balanced against significantly increased toxicity. Our own overall objective response rate and survival results are within the range reported in most other studies. An exception however is the so-called MIC (mitomyci-C, ifosfamide and cisplatin) schedule, one of the most active regimens so far reported with a response rate of 56% (Cullen *et al.*, 1988). It is unlikely that selection bias can entirely explain this difference, and a comparative trial would be useful in assessing the extent to which putative increased efficacy might outweigh the disadvantages of increased cost and toxicity of MIC in a palliative setting.

Meanwhile, we believe that MVP, a pragmatic and inexpensive chemotherapy regimen, should now be considered as an established therapy for symptom palliation in patients with metastatic NSCLC. An important practical point for clinical management is that symptom relief was usually achieved after one course of chemotherapy and nearly always after two. Palliative MVP chemotherapy should therefore be given as a therapeutic trial of two courses, and in general stopped after this if useful symptomatic relief has not been obtained. This prevents cumulative toxicity in patients failing to respond and targets more prolonged treatment at patients receiving real benefit. Furthermore, since objective response is nearly always associated with symptomatic relief, the latter can be used in the great majority of patients as evidence of clinical benefit without the need for elaborate and expensive restaging investigations including CT scans to confirm objective response.

Where does chemotherapy go next in NSCLC? An important area for study is primary (neoadjuvant) chemotherapy before surgery or radiotherapy for locally advanced (usually IIIB) disease. Several trials have already been reported, some showing significant survival benefit over radiotherapy alone (Dillman *et al.*, 1990), some showing benefit just below significance (LeChevalier *et al.*, 1991) and some showing no benefit (Morton *et al.*, 1991). Further trials are under way, including an important one using the MIC schedule (Cullen *et al.*, 1988), but results are not yet available. If survival improvement is established for primary chemotherapy in this context it is likely to be modest. Real clinical benefit will therefore also depend on cost-effectiveness and low treatment-associated morbidity. In this context, we believe that this pragmatic low-risk MVP schedule, with established efficacy and low toxicity, should now be assessed as primary (neoadjuvant) chemotherapy before radical radiotherapy for locally advanced NSCLC in a randomised trial.

**Acknowledgements**

We wish to thank our secretaries Julia Holborn and Alison Norton for their help in the preparation of this manuscript. We also acknowledge the close clinical collaboration for our consultant colleagues in chest medicine and surgery including, in particular, Dr Andrew Miller and Dr Rupert Courtenay-Evans (Mayday Hospital, Croydon); Dr Geoff Knowles (Kingston Hospital); Dr Nigel Cooke and Dr Paul Jones (St Helier Hospital, Carshalton); Dr Peter Mitchell-Heggs (Epsom District Hospital); Dr Paul Jenkins (East Surrey Hospital, Redhill); and Mr Norman Wright (St George's Hospital, Tooting).

**References**

CARTEI G, CARTEI F, CANTONE A, CAUSARANO D, GENCO G, TOBALDIN A, INTERLANDI G AND GIRALDI T. (1993). Cisplatin-cyclophosphamide-mitomycin combination chemotherapy with supportive care versus supportive care alone for the treatment of metastatic non-small cell lung cancer. *J Natl Cancer Inst.*, **85**, 794-800.

CORMIER Y, BERGERON, D., LE FORGE, J., LAVANDIER M, FOURNIER, M., CHENARD, J AND DESMEULES M. (1982). Benefits of polychemotherapy in advanced non small cell lung bronchogenic carcinoma. *Cancer*, **50**, 845-849.

CULLEN MH. (1993). The MIC regimen in non-small cell lung cancer. *Lung Cancer*, **9**, S81-S89.

CULLEN MH, JOSHI R, CHETIYAWARDANA AD AND WOODROFFE CM. (1988). Mitomycin, ifosfamide and cisplatin in non-small cell lung cancer: treatment good enough to compare. *Br. J. Cancer*, **58**, 359-361.

DILLMAN RO, SEAGREN SL, PROPERT KJ, GUERRA J, EATON WL, PERRY MC, CAREY RW, FREI EF AND GREEN MR. (1990). A randomized trial of induction chemotherapy plus high-dose radiation versus radiation alone in stage III non-small-cell lung cancer. *N. Engl. J. Med.*, **323**, 940-945.

DONNADIEU N, PAESMANS M AND SCULIER JP. (1991). Chemotherapy of non-small cell lung cancer according to disease extent: a meta-analysis of the literature. *Lung Cancer*, **7**, 243-252.

GANZ PA, FIGLIN RA, HASKELL CM, LA SOTO N AND SIAU J. (1989). Supportive care versus supportive care and combination chemotherapy in metastatic non-small cell lung cancer: does chemotherapy make a difference? *Cancer*, **63**, 1271-1278.

GRALLA RJ, CASPER ES, KELSEN DP, BRAUN DW, DUKEMAN ME, MARTINI N, YOUNG CW AND GOLBEY RB. (1981). Cisplatin and vindesine combination chemotherapy for advanced carcinoma of the lung: a randomized trial investigating two dosage schedules. *Annals of Internal Medicine*, **95**, 414-420.

HARDY JR, NOBLE T AND SMITH IE. (1989). Symptom relief with moderate dose chemotherapy (mitomycin-C, Vinblastine and cisplatin) in advanced non small cell lung cancer. *Br. J. Cancer*, **60**, 764-766.

JAAKKIMAINEN L, GOODWIN PJ, PATER J, WARDE P, MURRAY N AND RAPP E. (1990). Counting the costs of chemotherapy in a National Cancer Institute of Canada randomized trial in non-small cell lung cancer. *J. Clin. Oncol.*, **8**, 1301-1309.

KAASA S, LUND E, THORUD E, HATLEVOLL R AND HOST H. (1991). Symptomatic treatment versus combination chemotherapy for patients with extensive non-small cell lung cancer. *Cancer*, **67**, 2443-2447.

KAPLAN EL AND MEIER P. (1958). Non parametric estimation from incomplete observation. *J. Am. Stat. Assoc.*, **53**, 457-481.

KLASTERSKY J, SCULIER JP, RAVEZ P, LIBERT P, MICHEL J, VANDERMOTEN G, ROCHMANS P, BONDUELLE Y, MAIRESSE M, MICHIELS T, THIRIAUX J, MOMMEN P, DALESIO O AND THE EORTC LUNG CANCER WORKING PARTY (1986). A randomized study comparing a high and a standard dose of cisplatin in combination with etoposide in the treatment of advanced non small cell lung carcinoma. *J. Clin. Oncol.*, **4**, 1780-1786.

LE CHEVALIER T, ARRIAGADA RM QUOIX E, RUFFIE P, MARTIN M, TARAYRE M, LACOMBE-TERRIER M-J, DOUILLARD J-Y AND LAPLANCHE A. (1991). Radiotherapy alone versus combined chemotherapy and radiotherapy in nonresectable non-small-cell lung cancer: first analysis of a randomised trial in 353 patients. *J. Natl Cancer Inst.*, **83**, 417-423.

LUEDKE DW, EINHORN L, OMURA GA, SORMA PR, BARTOLUCCI AA, BIRCH R AND GREGO FA. (1990). Randomized comparison of two combination regimens versus minimal chemotherapy in non small cell lung cancer. A South Eastern Cancer Study Group Trial. *J. Clin Oncol.*, **8**, 886-891.

MILLER AB, HOOGSTRATEN B, STAQUET M AND WINKLER A. (1981). Reporting results of cancer treatment. *Cancer*, **47**, 207-214.

MORTON RF, JETT JR, McGINNIS WL, EARLE JD, THERNEAU TM, KROOK JE, ELLIOTT TE, MAILLIARD JA, NELIMARK RA, MAKSYMIUK AW, DRUMMOND RG, LAURIE JA, KUGLER JW AND ANDERSON RT. (1991). Thoracic radiation therapy alone compared with combined chemoradiotherapy for locally unresectable non-small cell lung cancer. *Ann. Intern. Med.*, **115**, 681-686.

MOUNTAIN CF. (1986). A new international staging system for lung cancer. *Chest*, **89**, 225-232.

RAPP E, PATER JL, WILLAN A, CORMIER Y, MURRAY N, EVANS WK, HODSON DI, CLARK DA, FELD R, ARNOLD AM, AYOUB JI, WILSON KS, LATREILLE J, WIERZBICKI RF AND HILL DD. (1988). Chemotherapy can prolong survival in patients with advanced non small cell lung cancer - Report of a Canadian multicentre randomized trial. *J. Clin. Oncol.*, **6**, 633-641.

RUCKDESCHEL JC, FINKLESTEIN DM, ETTINGER DS, CREECH RH, MASON BA, JOSS RA AND VOGL S. (1986). A randomised trial of the four most active regimens for metastatic non small cell lung cancer. *J. Clin. Oncol.*, **4**, 14-22.

SHINKAI T, SAIJO N, EGUCHI K, SASAKI Y, TOMINAGA K, SAKURAI M, SUGA J, MIYAOKA H, SANO T AND KEICHO N. (1986). Cisplatin and vindesine combination chemotherapy for non small cell lung cancer: a randomised trial comparing two dosages of cisplatin. *Jpn J. Cancer Res.*, **77**, 782-789.

SOUQUET PJ, CHAUVIN F, BOISSEL JP, CELLERINO R, CORMIER Y, GANZ PA, KAASA S, PATER JL, QUOIX E, RAPP E, TUMARELLO D, WILLIAMS J, WOODS BL AND BERNARD JP. (1993). Polychemotherapy in advanced non-small cell lung cancer: a meta-analysis. *Lancet*, **342**, 19-21.

VERONESI A, MAGRI MD AND TIRELLI U. (1988). Chemotherapy of advanced non-small cell lung cancer with cyclophosphamide, Adriamycin, methotrexate and procarbazine versus cisplatin and etoposide: a randomised study. *Am. J. Clin. Oncol.*, **11**, 566-571.

WOODS RL, WILLIAMS CJ, LEVI J, PAGE J, BELL D, BYRNE M AND KERESTES ZL. (1990). A randomised trial of cisplatin and vindesine versus supportive care only in advanced non-small cell lung cancer. *Br. J. Cancer*, **61**, 608-611.